# Nerve excitation using an amplitude-modulated signal with kilohertz-frequency carrier and non-zero offset

**DOI:** 10.1186/s12984-016-0171-4

**Published:** 2016-07-12

**Authors:** Leonel E. Medina, Warren M. Grill

**Affiliations:** Department of Biomedical Engineering, Duke University, Fitzpatrick CIEMAS, Room 1427, Box 90281, Durham, NC 27708-0281 USA; Department of Neurobiology, Duke University Medical Center, Durham, NC USA; Department of Surgery, Duke University Medical Center, Durham, NC USA; Department of Electrical and Computer Engineering, Duke University, Durham, NC USA

**Keywords:** Kilohertz frequency nerve stimulation, Transdermal amplitude modulated signal, Computational modeling, Nerve stimulation

## Abstract

**Background:**

Incorporating kilohertz-frequency signals in transcutaneous electrical stimulation has been proposed as a means to overcome the impedance of the skin, thereby reaching deeper nerves. In particular, a transdermal amplitude modulated signal (TAMS), composed of a 210 kHz non-zero offset carrier modulated by rectangular pulses, was introduced recently for the treatment of overactive bladder. However, the contribution of the components of TAMS to nerve fiber activation has not been quantified.

**Methods:**

We conducted in vivo experiments and applied direct stimulation to the sciatic nerve of cats and rats. We measured electromyogram and compound action potential activity evoked by pulses, TAMS and modified versions of TAMS in which we varied the size of the carrier.

**Results:**

Nerve fiber activation using TAMS showed no difference with respect to activation with conventional pulse for carrier frequencies of 20 kHz and higher, regardless the relative amplitude of the carrier. For frequencies lower than 20 kHz, the offset needed to generate half of the maximal evoked response decreased significantly with respect to the pulse. Results of simulations in a computational model of nerve fiber stimulation using the same stimulation waveforms closely matched our experimental measurements.

**Conclusion:**

Taken together, these results suggest that a TAMS with carrier frequencies >20 kHz does not offer any advantage over conventional pulses, even with larger amplitudes of the carrier, and this has implications for design of waveforms for efficient and effective transcutaneous stimulation.

## Background

Electrical stimulation of peripheral nerves is a treatment option for dysfunction of the lower urinary tract, chronic pain, epilepsy, and other neurological disorders [1]. In many cases, stimulation of peripheral nerves is delivered via an implanted device including an electrode in proximity to the nerve and a pulse generator. Transcutaneous electrical stimulation (TES) is a noninvasive alternative for peripheral nerve stimulation, in which an external electrode and stimulator deliver electrical currents across the skin, thereby reducing the risks and costs associated with implanted devices. Kilohertz frequency signals have been proposed as a means to overcome the high impedance of the skin, which declines by several orders of magnitude as the frequency of stimulation extends to the kHz range, and thereby reach deeper structure from the skin surface [2]. For example, in interferential currents therapy, the paths of two kHz currents applied to the skin cross and generate an amplitude modulated signal intended to stimulate peripheral nerves [3]. Recently, a novel waveform called Transdermal Amplitude Modulated Signal (TAMS) composed of a 210 kHz carrier modulated by rectangular pulses, was proposed for bladder control [4] and to treat overactive bladder [5]. In TAMS, the sinusoidal carrier is added to a traditional rectangular pulse of half the amplitude of the carrier, resulting in a sinusoidal burst (1 ms in duration) with a non-zero mean (offset).

Preclinical studies of TAMS for bladder control via TES of the pudendal nerve in cats [4, 6] concluded that TAMS modulated bladder activity similarly to direct pudendal nerve stimulation. Both inhibitory and excitatory effects on bladder activity were evoked, depending on the frequency of the modulating waveform, i.e., the rate at which the pulses were delivered, consistent with prior reports on direct nerve stimulation [7]. Elkelini et al. [8] applied sacral nerve stimulation in spinally transected rats and found that TAMS reduced detrusor overactivity presumably by indirectly inhibiting C-fiber activity. Further, clinical studies showed that TAMS improved the symptoms and quality of life of overactive bladder patients [5, 9]. However, the mechanisms of action of TAMS, which may differ substantially from conventional low-frequency stimulation using short-duration rectangular pulses, remain largely unexplored. In particular, there is no direct comparison of the efficacy and efficiency of TAMS to conventional pulses, and the contribution of each component of the TAMS waveform to nerve excitation has not been determined. Moreover, both experimental and computational modelling results suggest that the high-frequency carrier used in TAMS may not affect nerve excitation. Slovak et al. [10] applied TES to the forearm of healthy subjects using conventional pulses and TAMS and found no differences in sensation thresholds or EMG responses if the offset of TAMS was equal to the amplitude of the conventional pulses. Medina and Grill [11] found that TAMS and conventional pulses produced the same thresholds for activation of model nerve fibers, even though the voltages in the volume conductor, i.e., multiple layers of tissue simulating transcutaneous stimulation, were ~ 3–4 times larger at a certain depth from the surface for frequencies > 10 kHz as compared to lower frequencies.

**Fig. 1:**
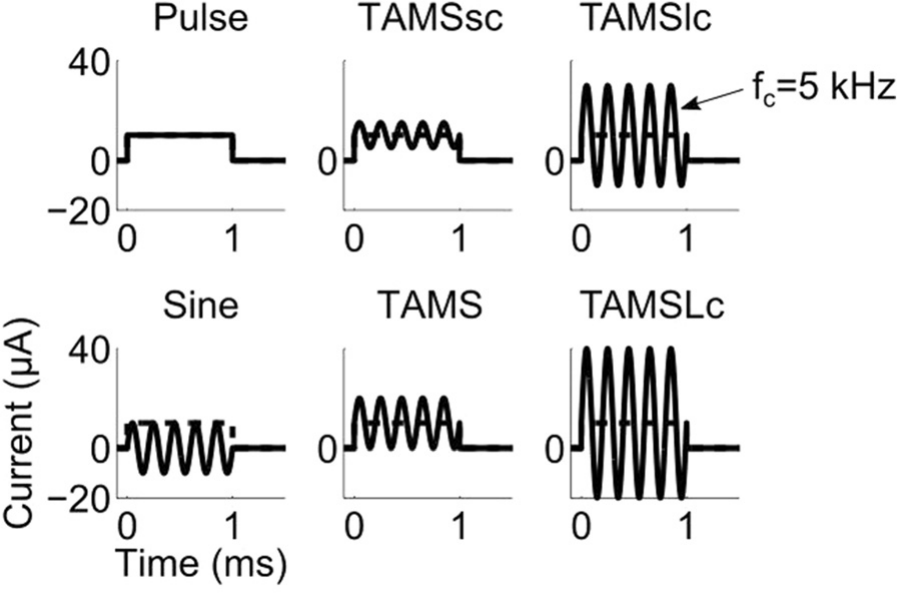
Stimulation waveforms. The plots illustrate the rectangular pulse, the sinusoidal burst, TAMS (α = 1) and the modified TAMS (α = 0.5, 2, and 3), respectively termed TAMS smaller-carrier (TAMSsc), TAMS larger-carrier (TAMSlc) and TAMS Largest-carrier (TAMSLc). All waveforms have an offset (K) equal to 1 (dashed lines) and a pulse width of 1 ms, except for the sinusoidal burst that has zero offset and amplitude 1

In the present study, we present a simple yet definitive experiment to quantify the effects of the carrier component of TAMS signal during direct nerve stimulation, i.e., bypassing the filtering properties of the skin and underlying tissue. We conducted in vivo experiments in rats and cats to measure electromyogram (EMG) and compound action potential (CAP) responses evoked by sciatic nerve stimulation using conventional pulses, TAMS, and modified versions of TAMS with different amplitudes of the carrier relative to the pulse component of the waveform (Fig. 1). Additionally, we implemented a computational model of nerve stimulation and quantified activation of model nerve fibers using the same stimulation waveforms. Thresholds were largely unaffected by the amplitude of the carrier if the carrier frequency was greater than 20 kHz, and it was the pulse component of the signal that generated nerve excitation. This quantitative assessment clarifies the contributions of the components of TAMS to nerve excitation and may be important for designing more efficient or effective waveforms for transcutaneous stimulation.

## Methods

### Stimulation waveforms

We quantified nerve excitation with five different stimulation waveforms: rectangular pulse, TAMS, and three modified versions of TAMS that differed in the amplitude of the carrier relative to the pulse component of the waveform (Fig. 1). In addition to these five waveforms, in the computational simulations we also used a sinusoidal burst with zero offset. Let *s(t)* denote the stimulation waveform and *f*_*c*_ denote the frequency of the carrier. TAMS and the modified versions of TAMS can be written as:

$$ s(t)=K\left(1+\alpha \sin \left(2\pi {f}_ct\right)\right)\kern2em 0\le t\le PW, $$

the rectangular pulse:

$$ s(t)=K\kern2em 0\le t\le PW, $$

and the sinusoidal burst:

$$ s(t)=A \sin \left(2\pi {f}_ct\right)\kern2em 0\le t\le PW $$

where *PW* is the pulse width, *K* is the signal offset, α is the amplitude of the carrier, and *A* is the amplitude of the sinusoidal burst. For TAMS α = 1, and we also used α = 0.5, α = 2 and α = 3, termed TAMS smaller-carrier (TAMSsc), TAMS larger-carrier (TAMSlc) and TAMS Largest-carrier (TAMSLc), respectively. We note that in TAMSsc the carrier amplitude is smaller than in TAMS, whereas in the other two modified versions the carrier amplitude is larger than in TAMS. In all experiments, we used *PW* = 1 ms, and *K* was varied to construct the input-output curves as nerve excitation as a function of stimulation amplitude.

### Computational model

**Fig. 2:**
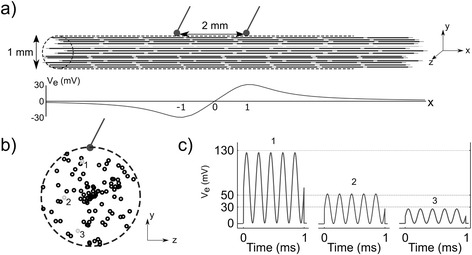
Modeling nerve stimulation. **a** Model nerve fibers were placed within and parallel to a cylinder of diameter 1 mm, and bipolar point source stimulation was applied along the edge of the cylinder. The bottom plot shows the potential generated by a pulse of 20 μA along the model fiber depicted in grey. **b** Transverse section of one example population of 100 nerve fibers randomly placed within the cylinder. **c** Potential generated by TAMS (carrier frequency: 5 kHz, offset: 25 μA) on the plane of one electrode for the three fibers indicated in (**b**)

We simulated extracellular stimulation of myelinated axons using the McIntyre-Richardson-Grill (MRG) cable model of a mammalian nerve fiber [12]. All axons had a diameter of 11.5 μm, representing the largest motor fibers found in the rat sciatic nerve [13]. We calculated the extracellular potentials generated by two point source electrodes of opposite polarity in an infinite anisotropic medium of conductivities 1/3 S/m in the longitudinal direction, i.e., parallel to the fibers, and 1/12 S/m in the transverse direction, i.e., perpendicular to the fibers [14]. We separated the point source electrodes by 2 mm as this separation was approximately equal to the separation of the poles of the hook electrodes used to deliver stimulation in the in vivo experiments. We positioned 100 axons containing 25 nodes of Ranvier within and parallel to a 1 mm diameter cylinder, with the electrodes placed along the edge of the cylinder (Fig. 2). The radial and azimuthal coordinates of the axons axes were drawn from a uniform distribution, and the middle node was randomly positioned with respect to the cathode, but no further than half the internodal length. We applied the potentials to all compartments of the fiber models using the extracellular mechanism in NEURON v7.3, and we neglected axon-to-axon (i.e., ephaptic) interactions. We used a simulation time of 3 ms and a time step of 1 μs, i.e., the sampling frequency was ten times the maximum frequency of the applied waveforms to avoid aliasing. We recorded the transmembrane potential at one end of the model axons to detect whether an action potential was elicited.

We constructed input-output (recruitment) curves using conventional pulses, sinusoidal bursts of zero mean, TAMS and modified TAMS, by increasing *K* (*A* for the sinusoidal burst) using a step of 5 μA until all model nerve fibers were activated. For the sinusoidal burst, TAMS and modified TAMS we used 7 different (carrier) frequencies: 1, 2, 5, 10, 20, 50 and 100 kHz. We defined I_50_ as the *K* (*A* for the sinusoidal burst) that activated half of the population, and the means and standard errors of these values across 8 different randomized populations of model nerve fibers were calculated.

### In vivo experiments

**Fig. 3:**
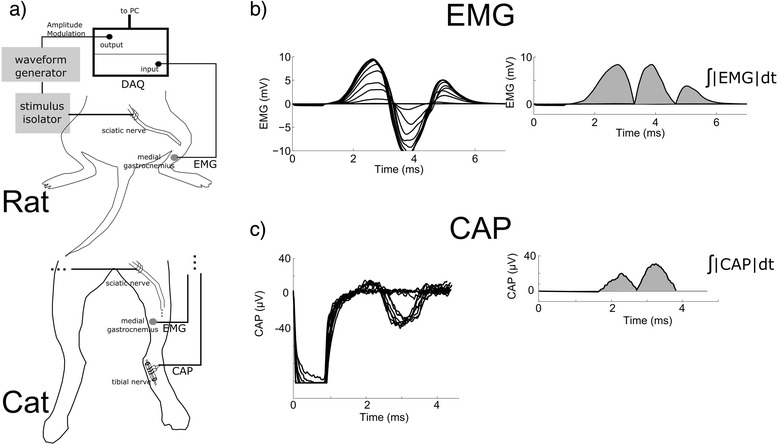
In vivo stimulation of rat and cat sciatic nerve. Stimulation was delivered through a bipolar hook electrode using a waveform generator controlled by computer. **a**. The evoked EMG signal was recorded from the medial gastrocnemius in rats and cats, and the evoked CAP was recorded from the tibial nerve in cats. **b**,**c** The plots show the evoked EMG (**b**) and CAP (**c**) responses using pulses of amplitudes 10–200 μA. The signals were rectified and integrated to construct input-output curves

All animal care and experimental procedures were approved by the Institutional Animal Care and Use Committees of Duke University. Experiments were performed on adult rats (*n* = 5) and cats (*n* = 3), and the procedures were acute and terminal. Rats were sedated by inhalation of isoflurane, and then anesthetized with urethane (1.2 g/kg, S.Q.), with supplemental doses (0.4 g/kg, S.Q.) given as required. Body temperature was regulated with the use of a thermostatically controlled water-circulated blanket. The sciatic nerve was exposed by dissection of the skin from the heel to the vertebral column and reflection of the biceps femoris muscle. A bipolar hook electrode was placed on the sciatic nerve near the sciatic notch to deliver stimulation. Electromyograms (EMG) from the medial gastrocnemius muscle were recorded using a pair of insulated fine stainless steel wires with exposed tips inserted percutaneously into the gastrocnemius musculature (Fig. 3).

Cats were sedated with acepromazine (0.3 mg/kg, S.Q.), and anesthesia was induced with ketamine HCl (15–35 mg/kg, I.M.) and maintained with alpha-chloralose (65 mg/kg, I.V., supplemented at 15 mg/kg, I.V.). Animals were intubated by tracheotomy and respired to maintain end tidal CO_2_ at 3-4 %. Body temperature was maintained with a water circulating heating pad, and arterial blood pressure was monitored using a catheter in the carotid artery. Fluids levels were maintained by administering 0.9 % saline with 8.4 mg/ml sodium bicarbonate and 5 % dextrose (15 ml/kg/h, I.V.). The sciatic nerve was exposed via an incision on the dorsal aspect of the thigh, and a bipolar hook electrode was placed on the nerve for stimulation. The tibial nerve was exposed via an incision medial to the distal portion of the calcaneal tendon, and a tripolar nerve cuff electrode (2 mm I.D.; 5 mm intercontact distance) was installed around the tibial nerve to record the compound action potential (CAP). Similar to the rat experiments, the EMG of the medial gastrocnemius muscle was recorded using a pair of stainless steel wires with exposed tips inserted percutaneously.

Stimulation was delivered through the hook electrodes using an arbitrary waveform generator (Agilent 33120A) and a linear voltage-to-current converter (A-M Systems 2200). The EMG and CAP signals was amplified (100–1,000 and 1,000–10,000, for EMG and CAP, respectively) and filtered (3 Hz – 10 kHz) using a low noise voltage amplifier (Grass Technologies P511 AC Amplifier), and recorded using the Powerlab hardware system and the LabChart software suite (ADInstruments Inc) with a sampling rate of 20 kHz.

Input-output curves of the rectified and integrated evoked EMG and CAP signals as a function of *K* were measured for conventional pulses, and for TAMS and the three modified versions of TAMS using seven carrier frequencies: 1, 2, 5, 10, 20, 50 and 100 kHz. We determined that 100 kHz was the maximum frequency that did not experience substantial attenuation by the voltage-to-current converter, and prior computational modeling results indicated that there is no significant difference in nerve fiber activation between TAMS with 100 kHz and 210 kHz carrier frequencies [11]. The input-output curve was recorded by delivering 1 ms duration sinusoidal bursts or pulses at 1 Hz and gradually increasing *K* in steps of 10–40 μA every 6–10 stimuli until the maximal response was evoked. In each experiment, the input-output curve for the conventional pulse was recorded first, followed by the remaining four waveforms, and the order of presentation of the seven carrier frequencies was randomized.

### Statistical analysis

We log-transformed the data and analyzed the effects of carrier frequency and carrier amplitude (α) on I_50_, which we defined as the *K* that produced 50 % of the maximum area of the rectified EMG or CAP response. We performed a separate ANOVA for the EMG and CAP data with I_50_ as the dependent variable and waveform type, carrier frequency (nested within waveform type), and species (in the case of EMG data) as the independent variables. The ANOVA for the EMG data revealed that species had no significant interactions with the other independent variables (*p* > 0.9 in both cases), and EMG data from both species were combined for further analysis. For tests that revealed significant differences among waveforms, *post hoc* paired comparisons were performed using Fisher’s protected least significant difference (FPLSD) test at a significance level of *p* < 0.05. Although data were log-transformed for analysis, data were plotted in the figures as average percent difference with respect to the pulse.

## Results

### Simulations in a computational model of peripheral nerve fiber stimulation

**Fig. 4:**
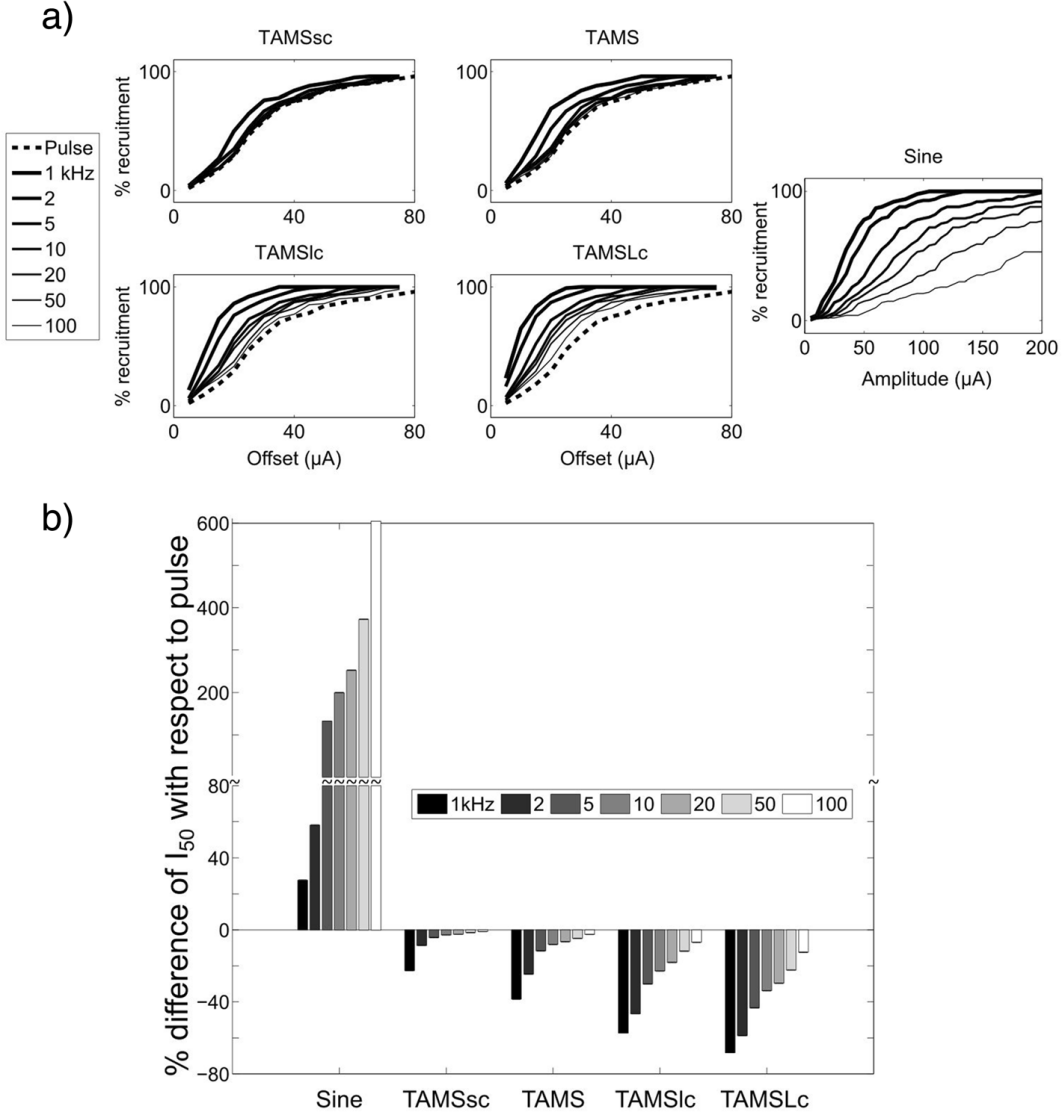
Simulation of nerve stimulation. **a** Recruitment curves for one sample population for all stimulation waveforms as a function of signal offset or signal amplitude. Each plot shows a different waveform using 7 (carrier) frequencies, except for the recruitment curve for the pulse, which was plotted in the TAMS graphs for comparison. **b** Percent difference of I_50_ with respect to pulse for sinusoidal burst, TAMS and modified TAMS using 7 (carrier) frequencies. Bars represent the average across 8 different axon populations. Error bars: SE

As the offset, *K,* (or the amplitude, *A,* for the sinusoidal burst) of the stimulation waveform was increased, the number of activated model nerve fibers increased monotonically for all stimulation waveforms (Fig. 4a). The carrier frequency of TAMS and modified TAMS, as well as the frequency of the sinusoidal burst, clearly affected the recruitment curves, and as the (carrier) frequency was decreased, the recruitment curve shifted to the left. We quantified the effect of the (carrier) frequency using I_50_, i.e., the signal offset (or amplitude) that activated 50 % of the population of model nerve fibers. Figure 4b shows the difference in I_50_ between the sinusoidal burst, TAMS and modified TAMS, and the rectangular pulse. All four TAMS versions had lower I_50_ compared to the pulse for all 7 carrier frequencies, and this difference increased for lower carrier frequencies. Further, for any given carrier frequency, I_50_ decreased more markedly as the size of the carrier increased, i.e., the largest difference with respect to the pulse was obtained for TAMSLc, followed by TAMSlc, TAMS and TAMSsc. On the contrary, the zero-offset sinusoid had much higher I_50_ than the rectangular pulse for all frequencies.

### In vivo measurements

**Fig. 5:**
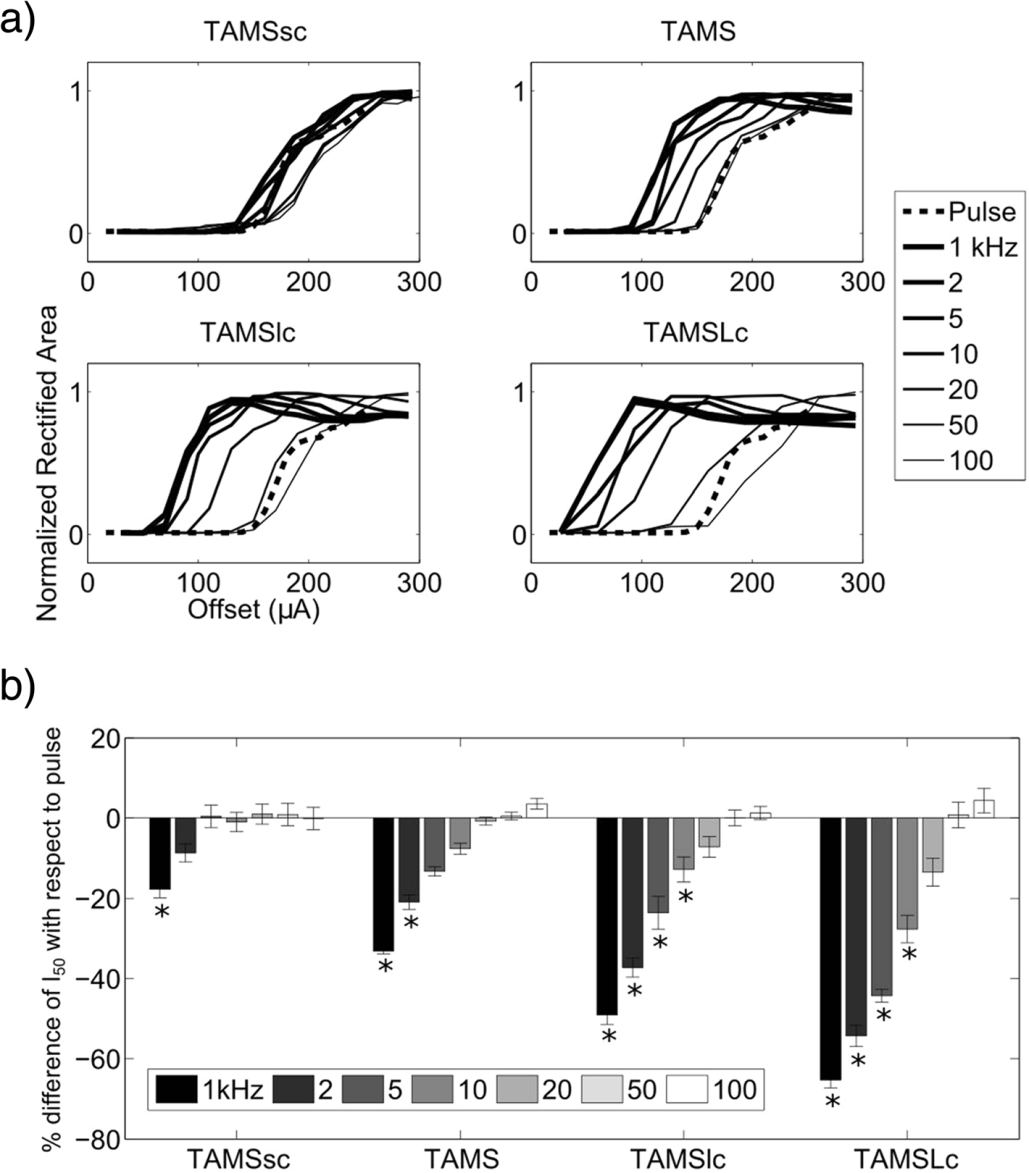
Evoked EMG activity. **a** Input-output curves measured in one animal for all stimulation waveforms. Each plot shows a different waveform using 7 carrier frequencies, except for the pulse, which was plotted in all 4 graphs. **b** Percent difference of I_50_ with respect to pulse for TAMS and modified TAMS using 7 carrier frequencies (mean ± SE, *n* = 8 combined data from rats and cats, * *p* < 0.05 difference with pulse)

**Fig. 6:**
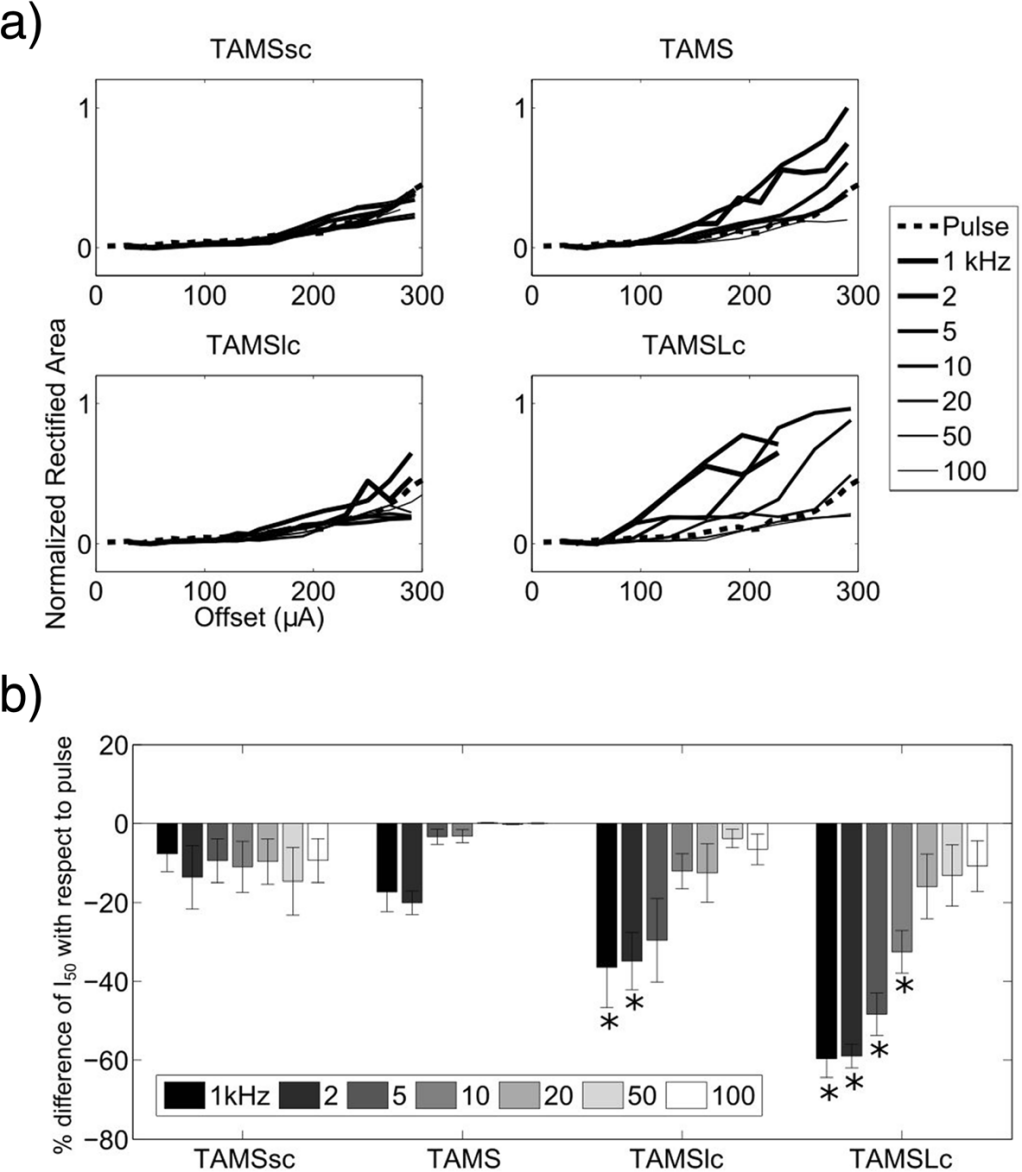
Evoked CAP activity. **a** Input-output curves measured in one animal for all stimulation waveforms. Each plot shows a different waveform using 7 carrier frequencies, except for the pulse, which was plotted in all four graphs. **b** Percent difference of I_50_ with respect to pulse for TAMS and modified TAMS for 7 carrier frequencies (mean ± SE, *n* = 3 cats, * *p* < 0.05 difference with pulse)

Figures 5 and 6 show the effects of stimulation waveforms on the in vivo EMGs and CAPs, respectively. The rectified EMG area increased as *K* was increased, and the sigmoidal input-output curves shifted to the left as the carrier frequency decreased (Fig. 5a). I_50_, i.e., the *K* that produced 50 % of the maximal rectified area depended on the carrier frequency. For the 1 kHz carrier, I_50_ averaged 142 ± 23.5 μA, 114 ± 18.8 μA, 94 ± 24.5 μA and 60 ± 11.5 μA (mean ± S.E.) for TAMSsc, TAMS, TAMSlc and TAMSLc, respectively, and for the 100 kHz carrier, I_50_ averaged 173 ± 29.0 μA, 175 ± 27.4 μA, 177 ± 29.8 μA and 188 ± 38.2 μA (mean ± S.E.), respectively. For the rectangular pulse, I_50_ averaged 168 ± 23.0 μA (mean ± S.E.). A two-way repeated measures ANOVA showed significant effects of waveform type and frequency (*p* < 0.0001 in both cases). *Post hoc* paired comparisons revealed significant differences (FPLSD, *p* < 0.05) between rectangular pulse, and TAMS and modified TAMS only for certain carrier frequencies (Fig. 5b). All four TAMS waveforms had significantly lower I_50_ compared to the rectangular pulse using the 1 kHz carrier, but none showed significant differences with the pulse for carrier frequencies higher than 10 kHz. Further confirmation of nerve activation as quantified by EMG responses was obtained with measurements of the CAP, which showed similar trends (Fig. 6). For the rectified area of the CAP, the input-out curves shifted to the left with frequency, as well. For the 1 kHz carrier, I_50_ averaged 265 ± 56.6 μA, 225 ± 13.5 μA, 187 ± 65.2 μA and 120 ± 35.7 μA (mean ± S.E.) for TAMSsc, TAMS, TAMSlc and TAMSLc, respectively. For the 100 kHz carrier, I_50_ averaged 262 ± 60.0 μA, 281 ± 41.3 μA, 268 ± 54.4 μA, 259 ± 62.8 μA (mean ± S.E.), respectively. For the rectangular pulse, I_50_ averaged 281 ± 41.0 μA. In this case, I_50_ for only TAMSlc of 1 and 2 kHz, and TAMSLc of 1, 2, 5 and 10 kHz, was significantly lower than for the rectangular pulse (FPLSD, *p* < 0.05).

## Discussion

We quantified the contributions of the carrier frequency and amplitude of TAMS to nerve fiber activation in vivo and using a computational model. Carrier frequencies of 20 kHz and higher (recall that the default carrier frequency of the original TAMS waveform was 210 kHz) did not contribute to excitation (i.e., activation was equivalent to the conventional pulse) regardless of the amplitude of the carrier, while lower carrier frequencies did contribute to excitation, as reflected by the substantial reductions in I_50_ relative to the conventional pulse without a carrier. Our simulation results closely matched our experimental measurements, and using a carrier frequency of 1 kHz, I_50_ in the model declined by 68, 57, 39 and 23 % with respect to the pulse, for TAMSLc, TAMSlc, TAMS and TAMSsc waveforms, respectively, while for the EMG measurements, the reductions were 65, 49, 33 and 18 %. Further, the model revealed much higher thresholds for the zero offset sinusoid as compared to the rectangular pulse, which supports the conclusion that the high frequency sinusoidal component of TAMS contributes little to nerve fiber excitation. Collectively, these results indicate that amplitude modulated kHz stimulation (>20 kHz) does not improve the efficacy or efficiency of electrical stimulation of peripheral nerves.

### Mechanisms of nerve fiber activation using TAMS

TAMS with carrier frequencies below 20 kHz had lower thresholds than conventional pulses. Specifically, for 1 kHz TAMS the reduction in I_50_ was 33 and 17 % for EMG and CAP responses, respectively. At this frequency, the 1 ms pulse comprises a single cycle of the carrier, and therefore the stimulation waveform may effectively act as a 500 μs pulse of amplitude twice the offset. In this case, the strength-duration (SD) relationship, which quantifies nerve fiber threshold as a function of pulse duration, can be used to predict the differences in threshold. The SD curve is commonly described using the equation *I*_*th*_ *= I*_*rh*_*(1 + T*_*ch*_*/PW)*, where *PW* is the pulse width, *I*_*rh*_ is the rheobase, i.e., threshold current for a very long PW, and *T*_*ch*_ is the chronaxie, i.e., PW to obtain a threshold of twice the rheobase [15]. The motor axons in the cat tibial nerve have a chronaxie of 0.53 ms [16]. It follows that if we consider the 1 kHz TAMS as a 0.5 ms pulse of double amplitude of the pulse, then the SD curve estimates a threshold for 1 kHz TAMS 33 % smaller than the conventional pulse, and this matches quite well our measurements of I_50_.

**Fig. 7:**
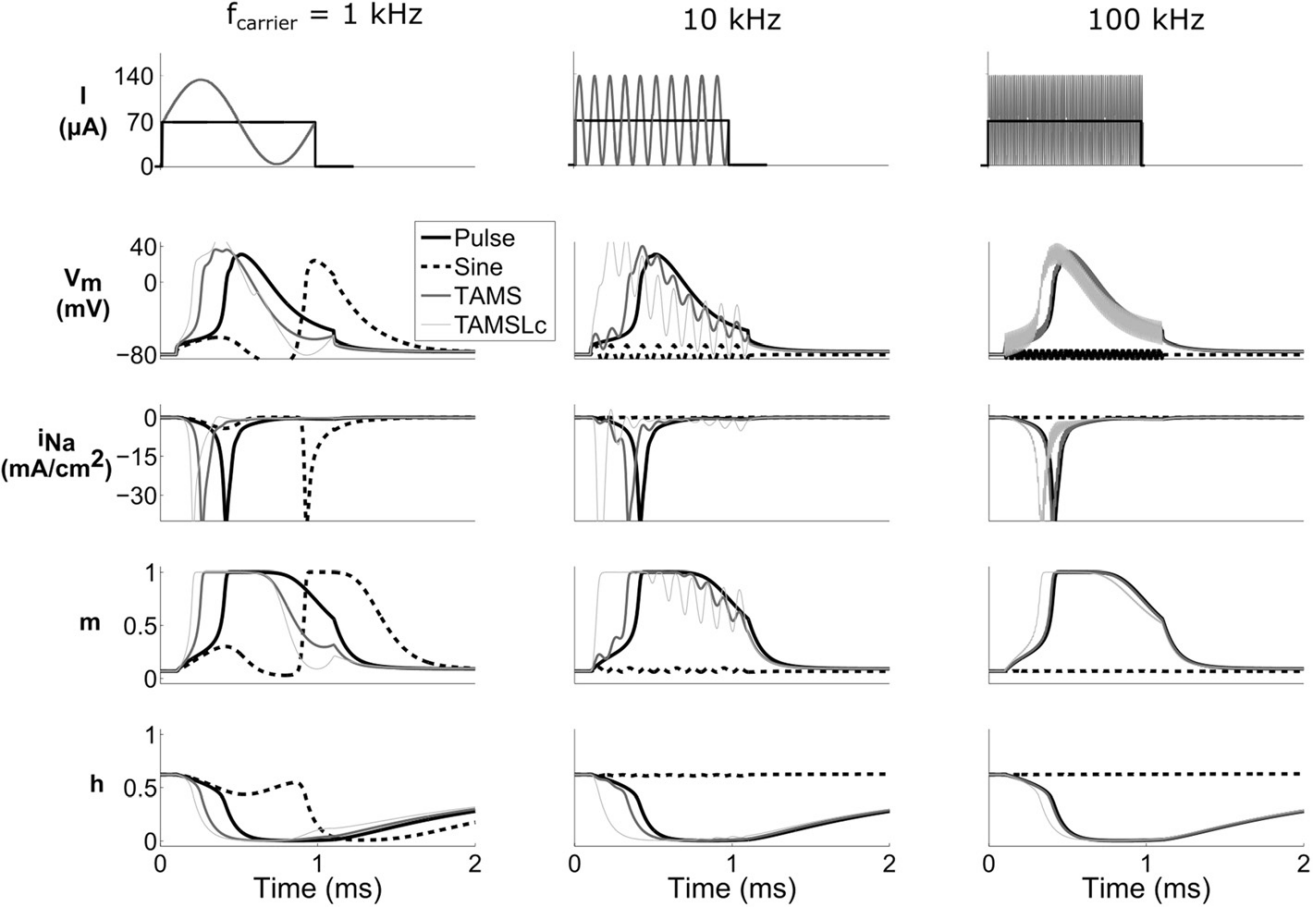
Membrane parameters during stimulation with pulse, sine, TAMS and TAMSLc. Each column represents a different (carrier) frequency, except for the pulse. The offset of the pulse, TAMS and TAMSLc was 70 μA, and the amplitude of the sine was 70 μA. An action potential was evoked in all cases, except for the 10 kHz and the 100 kHz sine. The pulse duration was 1 ms in all cases. The stimulation waveform (pulse and TAMS) is shown on the top row

To understand the mechanisms of nerve fiber activation using TAMS with carrier frequencies greater than 20 kHz, we recorded membrane parameters (transmembrane voltage, sodium current, and sodium conductance gating parameters) in the model nerve fiber during stimulation with TAMS, the conventional pulse, and sine waves of 1 ms duration. During extracellular stimulation using rectangular pulses, the time course of the gating parameters may differ depending on the amplitude and duration of the pulse, and at threshold, the action potential initiates near the end of the pulse [17]. The traces of the sodium current, I_Na_, were very similar for 100 kHz TAMS and for the rectangular pulse (third row, right panel of Fig. 7), i.e., the time courses of both currents overlapped, as it would be the case for two rectangular pulses of the same duration and well above threshold. The time course of the gating parameters, m and h, overlapped as well. Increasing the relative amplitude of the carrier, i.e., TAMSLc, only slightly altered the dynamics of the gating parameters, and the initiation of the action potential was shifted very slightly earlier in time. The membrane voltage showed only small oscillations at 100 kHz for the waveforms incorporating this frequency, and the zero-offset 100 kHz sine did not activate the axon. Therefore, the filtering of the membrane that passed the offset but attenuated the 100 kHz carrier, resulted in similar dynamics of the gating parameters, which explains nerve fiber excitation in this frequency range. On the other hand, for the 1 kHz carrier the dynamics of the gating parameters differed greatly. In this case, for a 1 ms duration, TAMS and the sinusoidal burst comprised only a single cycle of the sine wave. Further, TAMS roughly resembled a 500 μs monophasic pulse, and the sinusoidal burst, a biphasic pulse of 500 μs each phase. As a result, the action potential initiated at slightly different times for TAMS and TAMSLc, and somewhat later for the rectangular pulse, whereas for the sinusoidal burst axon activation occurred by the end of the hyperpolarizing phase.

Continuous zero mean ac signals in the 1 to 50 kHz range have been used for conduction block (for a review see [18]), but it is unclear whether amplitude modulated stimulation, as used in this study, can produce block. The onset of the kHz frequency block signal generates transient axon activation [18], and it is possible that in our experiments we observed a similar phenomenon, in which the transient phase of the stimulation waveform activated the axons. In their computational study, Bhadra et al. [19] simulated the onset phenomenon and observed distinct firing frequency responses to increasing signal amplitudes, from few action potentials to continuous firing, for both block and non-block signals. In our simulations, we observed only single action potentials for a wide range of amplitudes limited to the 1 ms duration of the pulses, and this range, i.e., up to few hundreds of μA, roughly matches the non-block amplitude range of Bhadra et al. using similar simulation conditions.

### Implications for transcutaneous electrical stimulation

TAMS was originally proposed for transcutaneous electrical stimulation and was intended to generate less attenuated voltages at deeper nerves. The idea was inspired by the experimental observation that the impedance of the skin declines by several orders of magnitude as the frequency increases to the kHz range [20]. Thus, high-frequency components, as the sinusoidal carrier in TAMS, may encounter less tissue impedance, thereby providing more current that may activate more deeply positioned nerves. As well, the impedance of the skin may be overcome by using very short duration pulses since shorter pulses have higher frequency content. In fact, the main lobe of the “sinc” function, i.e., the power spectrum of a single pulse, has a width that is inversely proportional to the pulse duration. For example, for a 10 μs pulse the power spectrum extends up to 100 kHz. Similar to kHz frequency sinusoidal bursts, the higher frequency components of a short pulse may overcome the skin impedance, but the main frequency component of the pulse is dc, regardless of its duration. While indeed our previous modeling work demonstrated less attenuation at depth of high frequency signals [11], the present findings indicate that high frequency components of the TAMS signal do not contribute to activation of nerve fibers, and this mitigates the possible benefit of deeper penetration. In our experiments, we bypassed the filtering effects of the skin by applying stimulation directly to the sciatic nerve, and manipulated the amplitude of the carrier. By means of this manipulation, we mimicked a scenario in which the filtering by the skin (and other intervening tissue) attenuates the (dc) offset much more so than the kHz frequency carrier, which we increased by as much as three times the size of the offset (i.e., TAMSLc). Since we observed no contribution of carriers of 20 kHz and above to nerve fiber activation, any reduction of carrier attenuation in TES will not affect nerve fiber excitation, as well.

Our results are in agreement with those of Slovak et al. [10] who found no differences in either motor thresholds or sensation thresholds between TAMS and conventional pulses. In their experiment, the authors applied current-controlled TES to the forearm of ten subjects and recorded the electrode-skin impedance, the motor threshold as identified using the EMG, and the first reported sensation upon increasing stimulation amplitude. Since these measures showed no differences between TAMS and rectangular pulses, the authors concluded that it is unlikely that TAMS provides any efficacy improvement over conventional stimulation. In clinical applications, it is yet to be determined whether TAMS can produce less skin discomfort than conventional pulses. TAMS improved the symptoms of patients with overactive bladder via stimuli delivered over the sacrum that presumably activated the sacral roots [5]. However, TAMS was not compared against conventional pulses in this clinical trial, and therefore it is unclear whether patients would experience less discomfort with TAMS as compared to conventional pulses. Importantly, our results suggest that subjects would not experience less discomfort with TAMS because for any given signal offset, TAMS (>20 kHz) produced the same level of nerve fiber activation as the rectangular pulse, regardless the amplitude of the carrier. It is very unlikely that the more superficial sensory fibers conveying pain information would respond differently. In fact, our modeling results [11] suggest that there is no inversion of the current-distance relationship for TAMS, and thus TAMS and the rectangular pulse would activate the same fibers at any given distance from the skin electrode. Slovak et al. measured sensory thresholds as well, and found no difference between TAMS and rectangular pulse. These results also support the idea that discomfort would be the same in sacral transcutaneous stimulation.

In their animal studies, Shen et al. [4] used voltage-controlled TES, and therefore voltage- versus current-controlled stimulation may at least partially explain the purported benefits of TAMS for bladder control by transcutaneous pudendal nerve stimulation. For voltage-controlled stimulation, the current amplitude that activates the nerve fibers depends on the frequency of stimulation because the impedance of the electrode-skin interface, which also depends on time, determines the voltage drop across the interface [21]. In contrast, for current-controlled stimulation, the amplitude of the current delivered to the tissue does not depend on frequency. In addition, the voltage and current transients for both types of stimulation were measured experimentally using pulses, and showed great differences [22]. Similarly, for TAMS, the transient of the delivered current for voltage- and current-controlled stimulation may also differ greatly, resulting in differences in the filtered signal that stimulates the nerve and, consequently, differences in nerve fiber activation. Therefore, the tissue filtering effects in both types of stimulation may differentially affect nerve activation. However, our results suggest that such filtering plays a minimal role in nerve fiber excitation using TAMS (>20 kHz) because changing the relative amplitude of the carrier (as a proxy for filtering) had no effect on nerve fiber excitation. As well, prior modelling work showed that nerve fiber thresholds for 100 kHz TAMS and conventional pulses were the same for both voltage- and current-controlled stimulation [11]. Finally, TAMS will not improve the efficiency of stimulation, as the addition of high-frequency carriers on top of the offset only increases the delivered energy. Instead, transcutaneous stimulation may be more efficient using other stimulation waveforms that reduce energy and/or power required for peripheral nerve stimulation [23].

### Limitations

One of the limitations of our study was not including frequencies higher than 100 kHz. In the original formulation of TAMS, Shen et al. [4] used a carrier of 210 kHz, but it is unclear how this particular frequency was selected. The changes in skin conductivity and impedance between 100 and 200 kHz are minimal [20, 24], and thus it is unlikely that we would observe different contributions to excitation between these frequencies. Further, a computational model of TES showed no difference in thresholds for axon activation using TAMS of 100 and 200 kHz [11].

The axon model that we used in this study, the MRG model, has been used to model conduction block with kHz frequency sinusoidal signals, and the results matched well the experimental data [19]. However, the model has not been thoroughly validated for activation of nerve fibers using kHz signals. For such validation, the model may need to be updated. Firstly, the capacitance of the axon membrane exhibits dispersion, and its conductance declines by a factor of two between dc and 100 kHz [25]. Howell et al. [26] quantified the effects of dispersive capacitance by incorporating a frequency-dependent membrane capacitance, c(f), in the MRG model. The threshold for generation of a single action potential using kHz sinusoidal signals applied for 10 ms declined by up to 14 % after incorporating c(f) in a 11.5 μm axon. Thus, it is possible that incorporating c(f) would further reduce thresholds in our simulations, but since we observed a greater effect of the offset, such reduction should be marginal. Finally, the MRG model does not include all ionic conductances present in human peripheral myelinated nerve fibers (e.g., [27]), and the mathematical equations describing the dynamics of ion channel were constructed based on experimental observations with low-frequency signals. Therefore, further investigation is needed to determine if the number and type of channels and their responses to kHz signals can accurately describe axon activation using TAMS and other high-frequency signals.

## Conclusion

We conducted in vivo experiments and computational simulations to quantify the effects of an amplitude-modulated signal with a kHz frequency sinusoidal carrier and a non-zero 1 ms pulse offset, called TAMS, on nerve fiber activation. TAMS with carrier frequencies of 20 kHz or greater generated the same activation as a conventional rectangular pulse, even after increasing the size of the carrier. Therefore, the addition of high-frequency carriers to the stimulation signal did not improve efficacy or efficiency in peripheral nerve stimulation, and in particular in TES, in which kHz signals have been tested as a means to overcome the skin impedance.

## Abbreviations

CAP, compound action potential; EMG, electromyogram; FPLSD, Fisher’s protected least significant difference; I_rh_, rheobase; MRG, McIntyre-Richardson-Grill; PW, pulse width; SD, strength-duration; TAMS, transdermal amplitude modulated signal; TAMSlc, TAMS larger-carrier; TAMSLc, TAMS Largest-carrier; TAMSsc, TAMS smaller-carrier; T_ch_, chronaxie; TES, transcutaneous electrical stimulation
